# Simulation of shear bands with Soft PARticle Code (SPARC) and FE

**DOI:** 10.1007/s13137-016-0091-2

**Published:** 2017-01-02

**Authors:** Barbara Schneider-Muntau, Chien-Hsun Chen, S. M. Iman Bathaeian

**Affiliations:** Division of Geotechnical and Tunnel Engineering, University of Innsbruck, Technikerstr. 13, 6020 Innsbruck, Austria

**Keywords:** Shear band, Barodesy, Soft PARticle Code (SPARC), Finite Element method, Biaxial test, 35D35, 35Q74, 65D05, 65M99, 74D10, 74C15

## Abstract

The aim of this paper is to numerically investigate the development, thickness and orientation of shear bands, in biaxial test with two approaches towards solving problems of continuum mechanics, namely the meshless “Soft PARticle” method and the mesh based Finite Element method. Soft PArticle Code (SPARC) is a straightforward collocation numerical method based on strong formulation, in which a first order polynomial basis is adopted for the evaluation of spatial derivatives in partial differential equations. A novel nonlinear constitutive model— barodesy for clay, is adopted in this study. The biaxial test, which involves homogeneous, and later inhomogeneous localized deformation is simulated using the Soft PArticle Code and the Finite Element method. The inclination and thickness of the shear bands are evaluated and analysed with the earlier experimental, theoretical and numerical investigations. Furthermore, simulation results are compared and presented to demonstrate the advantages and limitations of SPARC in comparison to FE method.

## Introduction

Meshless methods have drawn attention in the past decades due to their simplicity in numerical formulation and ability of modeling large deformations. Compared to mesh based methods such as FE methods, a true meshless method maintains neither the connectivity between the nodes nor uses any background mesh, therefore, no mesh is required. In addition, the calculation of spatial derivatives in partial differential equations is achieved by means of the information (the parameter of interest e.g. stress or velocity and the position vector) carried on by each particle. The point collocation method (Aluru 2000) and finite point methods (Onate et al. 2001), for instance, belong to this category. These methods use a finite support domain (small in comparison to the whole study domain) to approximate the spatial derivatives, and hence belong to collocation methods, which result in significant reduction of computational efforts (Zhang et al. 2000). Thereafter, the governing equations are solved, meaning that such a formulation is “strong”.

It has been shown that although SPARC adopts basic polynomial linear approximation method, it is capable of modeling strain localization (Chen 2005). In addition, the formulation for stress boundary condition is straightforward.

Finite element (FE) methods have been developed since the 1950’s. They are often regarded as benchmarks for new methods (Liu et al. 2006b, a) due to their robustness and accuracy. FE uses the weak formulation to solve the underlying differential equations. In this paper, the simulation results of a biaxial test using SPARC are compared with those obtained with an FE method. Two sets of biaxial test on overconsolidated specimen, once with a weak zone and once homogeneous are modeled. The weak zone guarantees the occurrence of inhomogeneous deformation and localization of deformation after the homogeneous deformation and manifests strain localization. Strain localization in real samples has e.g. been captured by means of x-ray on test samples (Kolymbas 1972). Modeling of strain localization is a challenging task, it can thus be used to test the capability of numerical simulations and serves as a good numerical example for comparison herein.

In our simulations, barodesy for clay (Medicus and Fellin 2015) is used as constitutive model. Barodetic constitutive models (Kolymbas 2012a, b; Medicus and Fellin 2015) take into account the influence of stress state $\mathbf {T}$ and void ratio *e* of the material. Their incremental stiffness (Kolymbas 1999) imposes convergence difficulties (Fellin and Ostermann 2002) especially at the occurrence of strain localization. This paper is organized as follows: in Sect. 2, the framework of SPARC is briefly explained, Sect. 3 provides some information regarding the adopted constitutive model barodesy for clay, in Sect. 4 the conventional soil parameters as defined by Mohr–Coulomb are obtained, Sect. 5 presents the simulation setups and results using SPARC and FE method and in Sect. 6 the acquired shear bands are investigated and compared. Conclusions are given in Sect. 7.

## SPARC framework

Soft PArticle Code is a straightforward framework for numerical simulation based on collocation strong formulation (Chen 2005; Ostermann et al. 2013). Continuum is presented using particles that carry physical information such as position vector $\mathbf x$, velocity vector $\mathbf {v}$, Cauchy stress tensor $\mathbf {T}$, void ratio *e* and etc. The equilibrium equation for each particle reads:

1 $$\begin{aligned} \text {div}^{t+\varDelta t} \mathbf {T}+ \rho {\mathbf {g}} = \mathbf {0} \end{aligned}$$

Since in this study the body force induced by gravitational acceleration *g* is negligible, *g* is set to zero in our simulations.

**Fig. 1:**
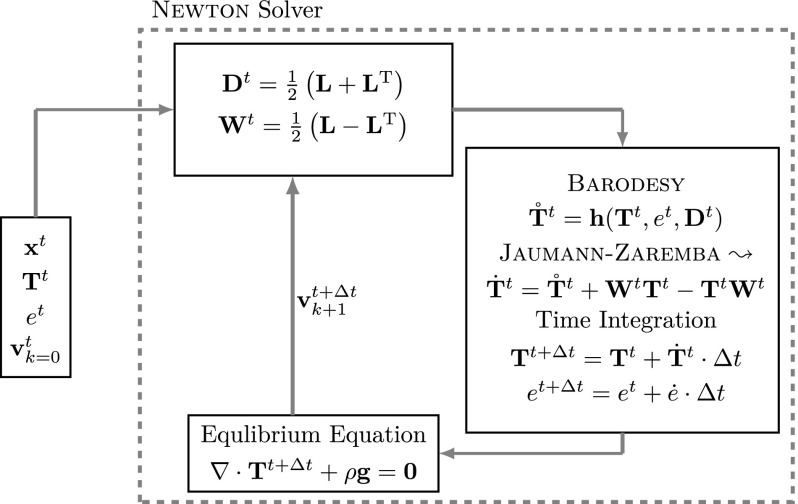
Framework of SPARC

The velocities of particles are the unknowns in SPARC. An initial guess of the velocity field $\mathbf {v}_{k=0}$ is considered as the start point of the solution finding process (see Fig. 1). The velocity gradient $\mathbf {L}=\text {grad}^t \mathbf {v}_{k=0}$ is then evaluated by means of “first order polynomial” interpolation. We then obtain the stretching tensor $\mathbf {D}$ and the spin tensor $\mathbf {W}$, which are the symmetric and anti-symmetric part of $\mathbf {L}$ as shown in Fig. 1. The objective rate of the stress tensor ${\mathbf {T}}^t$ at time-step *t* is evaluated by the constitutive model “barodesy for clay”,

2 $$\begin{aligned} {\mathbf {T}}^t=B(\mathbf {T}^t,\mathbf {D},e^t) \end{aligned}$$

which is a function of the current stress state $\mathbf {T}^t$, $e^t$ and $\mathbf {D}$ of the particle under evaluation. Due to highly nonlinear nature of barodesy for clay, the fourth-order Runge–Kutta integration is applied to obtain a more accurate objective stress rate ${\mathbf {T}}^{t}$ [see Chen (2005)]. Since the void ratio *e* is also one of the variables affecting the stress rate, the void ratio is also updated in every sub-step of the Runge–Kutta fourth-order by calculating the rate of void ratio

3 $$\begin{aligned} \dot{e}=(1+e) \ \text{ tr }\,\mathbf {D}, \end{aligned}$$

where $\text{ tr }\,\mathbf {D}$ is the sum of the diagonal components of the stretching tensor. Application of Jaumann–Zaremba objective stress rate yields the stress rate $\dot{\mathbf {T}}$,

4 $$\begin{aligned} \dot{\mathbf {T}}^{t} = {\mathbf {T}}^{t}+\mathbf {W}\mathbf {T}^{t}-\mathbf {T}^{t}\mathbf {W}\end{aligned}$$

and finally the stress state and void ratio are updated as follows:

5 $$\begin{aligned} \mathbf {T}^{t+\varDelta t}&=\mathbf {T}^t+\dot{\mathbf {T}}\varDelta t \end{aligned}$$

6 $$\begin{aligned} e^{t+\varDelta t}&=e^t + \dot{e} \varDelta t. \end{aligned}$$

The above mentioned algorithm is applied to all particles and the updated stress tensor $\mathbf {T}^{t+\varDelta t}$ and void ratio $e^{t+\varDelta t}$ for all particles are obtained. The equilibrium equation Eq. (1) can be built once the $\mathbf {T}^{t+\varDelta t}$ has been calculated. The non-linear system of equations consisting of Eq. (1) is solved by Newton method in order to determine the unknown velocities $\mathbf {v}^{t+\varDelta t}_{k+1}$, where *k* is the iteration of the Newton solver until the solution which satisfies the Eq. (1) is obtained. Once the solution $\mathbf {v}^{t+\varDelta t}$ is available, the position of the particles are updated,

7 $$\begin{aligned} \mathbf x^{t+\varDelta t}=\mathbf x^t + \mathbf {v}^{t+\varDelta t} \cdot \varDelta t \end{aligned}$$

and the solution of the velocity field from the previous time step is used as the first guess for the next velocity field time step.

Since SPARC is a collocation method, the Jacobian is sparse. In case of the presence of stress boundary conditions, such as the hydrostatic pressure applied on the biaxial test specimen, the kinematic boundary condition

8 $$\begin{aligned} \mathbf {T}^{t+\varDelta t}\mathbf {n}^{t+\varDelta t}-\mathbf {n}^{t+\varDelta t} p=\varvec{0} \end{aligned}$$

is applied, where $\mathbf {n}$ and *p* are the surface normal vector and pressure, respectively. Replacing Eq. (1) as the governing equation for boundary particles subject to surface pressure *p*.

It can be seen that the spatial derivatives of the velocity field and stress field (i.e. $\text {grad}\mathbf {v}$ and $\text {div}\mathbf {T}$) and the unit vector normal to the free surface must be evaluated to obtain the partial differential equations (PDE) in Eq. (1) or in Eq. (8). To achieve this, the linear polynomial approximation method is adopted. The following first order polynomial for *m*-dimensional problems is used:

9 $$\begin{aligned} \hat{f} = \sum _{j}^{m}a_j x_j \end{aligned}$$

where $\hat{f}$ is an approximation to the parameter of interest *f*. Its coefficients $a_j$ are computed in a least-squares sense, benefiting from the information *f* carried by the adjacent particles, the so-called neighbors. Here comes a 2-dimensional example for the evaluation of the coefficients $a_j$ for a particle with index *i*:10
11where $n_n$ denotes the total number of neighbors of particles *i*; the subscript denotes the corresponding physical property belonging to particle *i*; subscripts 1, 2, dots and $n_n$ denote the index of neighbors of particles *i*; $F_{(i)}$ and $\varvec{\mathcal {X}}_{(i)}$ are matrices consisting of the parameter of interest *f* and the position vectors $\mathbf x$ stored at neighbors of particle *i*, respectively. The coefficients can thus be obtained with12Herein, neighbors of the under-evaluation particle, are particles, which lie within a cut-off radius *r* of that particle. Note that the particle under evaluation is also counted as its own neighbor. The order of the neighbors in Eq. (11) is irrelevant, but the order of Eq. (10) must correspond to Eq. (11). Once the coefficients of $\hat{f}$ are obtained, the partial derivatives of $\hat{f}$ are also obtained: $\frac{\partial \hat{f}}{\partial x_i}=a_i$. If  is not a square matrix, pseudo-inverse is utilized to compute .

In SPARC, the logarithmic definition of strain is implemented so as to account for large deformations. It is believed that although the homogenized deformation of the whole specimen can be considered small enough to fit to the description of infinitesimal strain theory, the shear deformation in the shear bands is large enough to invalidate the theory of infinitesimal strain. The complete algorithm of how SPARC works is summarized in Fig. 1.

## Constitutive model

Barodesy is a constitutive model which can be seen as a further development of Hypoplasticity, which does not include notions of elastoplasticity such as yield and plastic potential functions. Barodesy, pioneered by Kolymbas (2012c) was first developed for sand and then developed for clay (Medicus and Fellin 2015). It is formulated in stress-rates (${\mathbf {T}}$) as a function of the stretching tensor ($\mathbf {D}$), the actual stress state $\mathbf {T}$ and the void ratio *e*:

13 $$\begin{aligned} {\mathbf {T}}=f(\mathbf {D},\mathbf {T},e). \end{aligned}$$

It is based upon the two rules by Goldscheider (1976), the so-called proportional paths and the asymptotic soil behavior. Deformations with proportional strain paths result in stress paths which approach a proportional stress path. Proportional strain paths are, for example, oedometric or triaxial compression. Furthermore, common concepts of soil mechanics, as e.g. barotropy, pyknotropy or critical state concept are considered (Kolymbas and Medicus 2016; Medicus et al. 2016). The complete equation of “barodesy for clay” reads,

14 $$\begin{aligned} {\mathbf {T}}=c_3 |\mathbf {T}|^{c_4}\cdot (f\mathbf {R}^0 + g\mathbf {T}^0)\cdot |\mathbf {D}| \end{aligned}$$

where $\mathbf {R}$ is the function, which links proportional strain paths to proportional stress paths and includes the *stress-dilatancy* relation. It must be noted that the constants $c_i$ are dimensionless. The ($\mathbf {R}^0$ and $\mathbf {T}^0$) are the normalized tensors^1^.

15 $$\begin{aligned} \mathbf {R}&=-\exp \left( \alpha \mathbf {D}^0\right) \quad \text {with}\;\; \alpha =\frac{\ln K}{\sqrt{3/2-{\text{ tr }\,\mathbf {D}^0}^2/2}} \end{aligned}$$

16 $$\begin{aligned} K&=1-\frac{1}{1+c_1(m-c_2)^2}\quad \text {with} \;\; m=\frac{-3\text{ tr }\,\mathbf {D}^0}{\sqrt{6-2{\text {tr}\mathbf {D}^0}^2}} \end{aligned}$$

where $\text{ tr }\,\mathbf {D}^0$ is the sum of diagonal components of the normalized stretching tensor and is a measure of dilatancy.

Functions *f* and *g* are scalar functions that incorporate asymptotic states, critical states, the influence of stress level (barotropy) and density (pyknotropy),

17 $$\begin{aligned} f&=c_6\cdot \beta \cdot \text{ tr }\,\mathbf {D}^0 - \frac{1}{2} \end{aligned}$$

18 $$\begin{aligned} g&=(1-c_6)\cdot \beta \cdot \text{ tr }\,\mathbf {D}^0 + \left( \frac{1+e}{1+e_c}\right) ^{c_5} -\frac{1}{2} \end{aligned}$$

with the critical void ratio $e_c$

19 $$\begin{aligned} e_c=\exp \left( N-\lambda ^* \ln \frac{2p}{\sigma ^*}\right) -1, \end{aligned}$$

where $\sigma ^*$ is the reference pressure (equal to 1 kPa) and

20 $$\begin{aligned} \beta&=-\frac{1}{c_3\varLambda }+\frac{1}{\sqrt{3}}2^{c_5\lambda ^*}-\frac{1}{\sqrt{3}} \end{aligned}$$

21 $$\begin{aligned} \varLambda&=-\frac{\lambda ^* - \kappa ^*}{2\sqrt{3}}\text{ tr }\,\mathbf {D}^0 + \frac{\lambda ^* + \kappa ^*}{2}. \end{aligned}$$

The constants $c_i$ are determined by means of soil parameters as follows,

$$\varphi _c$$:
critical friction angle
*N*: ordinate intercept of the isotropic normal compression line (NCL) in the $\ln p - \ln (1+e)$ plot
$$\lambda ^*$$:
is the slope of the NCL
$$\kappa ^*$$:
is the slope of unloading line under isotropic compression in $\ln p - \ln (1+e)$ plot

The calibration of constants for Dresden clay are found in Table 1.

**Table 1:** Determination of the constants for Dresden clay with $\varphi _c=35^{\circ }$, $N=0.622$, $\lambda ^*=0.038$ and $\kappa ^*=0.008$

Constants	Relation	Values for Dresden clay
$c_1$	$\frac{1-\sin \varphi _c}{2c_2^2\sin \varphi _c}$	0.028
$c_2$	$-\frac{3\sqrt{2}+3}{2}$	$-$3.6213
$c_3$	$\frac{-\sqrt{3}/\lambda ^*+\sqrt{3}/\kappa ^*}{2^{c_5\lambda ^*}+\left( \frac{1}{500}\right) ^{c_5\lambda ^*}}-2$	$-$356.408
$c_4$	1	1
$c_5$	$\frac{1+\sin \varphi _c}{1-\sin \varphi _c}$	3.69
$c_6$	$\frac{1}{2\left( \frac{-\sqrt{3}}{c_3\kappa ^*}+2^{c_5\lambda ^*}-1\right) }$	0.704

All parameters can be obtained from a consolidated undrained triaxial compression test. Readers are referred to Medicus and Fellin (2015) for more profound details on calibration and structure of “barodesy for clay”.

### Critical state in barodesy

**Fig. 2:**
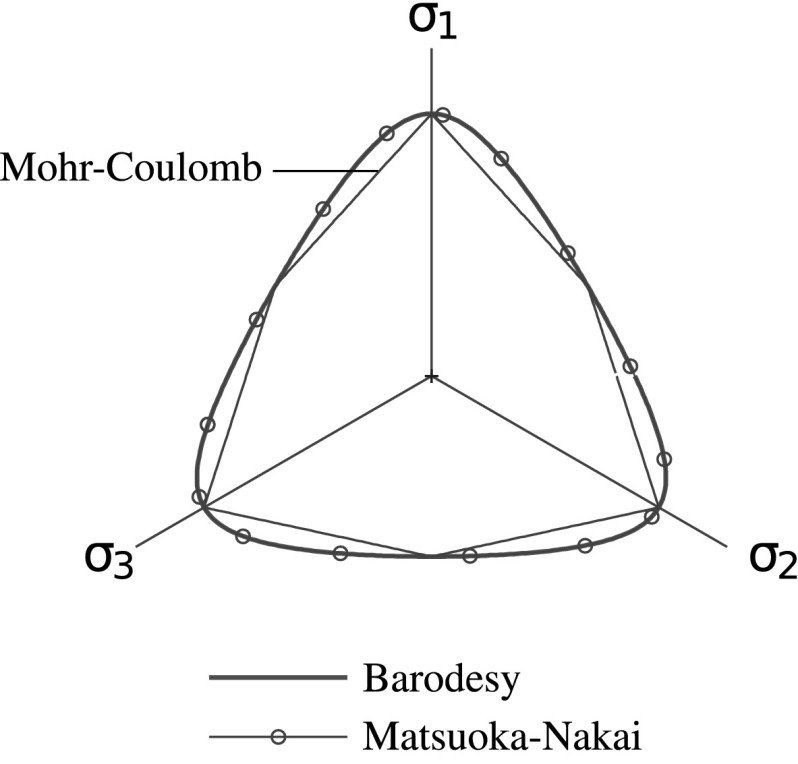
Coincidence of the locus of critical states of barodesy with Matsuoka-Nakai failure criterion. Mohr–Coulomb’s failure surface is added for comparison [from Medicus (2016, p. 57)]

The $\mathbf {R}$ function of barodesy has the major contribution in defining the critical state behavior, all stress directions $\mathbf {R}^0$ for isochoric ($\text{ tr }\,\mathbf {D}=0$) form a fan in the principal stress state (see Fig. 2). This locus coincides practically with the failure criterion of Matsuoka–Nakai for granular materials, which is defined as:

22 $$\begin{aligned} \frac{I_1 I_2}{I_3}=K_{MN} \end{aligned}$$

where $I_1=\text{ tr }\,\mathbf {T}$, $I_2=(I_1^2-\text {tr}\mathbf {T}^2)/2$ and $I_3=\det \mathbf {T}$ are the first, second and third invariants of the stress tensor, respectively. The material parameter $K_{MN}$ is a function of the critical state friction angle $\varphi _c$:

23 $$\begin{aligned} K_{MN}=\frac{9-\sin ^2 \varphi _c}{1-\sin ^2 \varphi _c}. \end{aligned}$$

Fellin and Ostermann have proven in Fellin and Ostermann (2013) that the deviation of the locus defined by barodesy from the one defined by the Matsuoka-Nakai criterion is less than $0.12 \%$ for a critical state friction angle of less than $40^{\circ }$. The barodetic model has been implemented in Abaqus as a user subroutine Umat based on the hypoplastic formulation presented in Fellin and Ostermann (2002). The void ratio is an additional state and output variable in the Umat.

## Mohr–Coulomb vs. barodesy

**Fig. 3:**
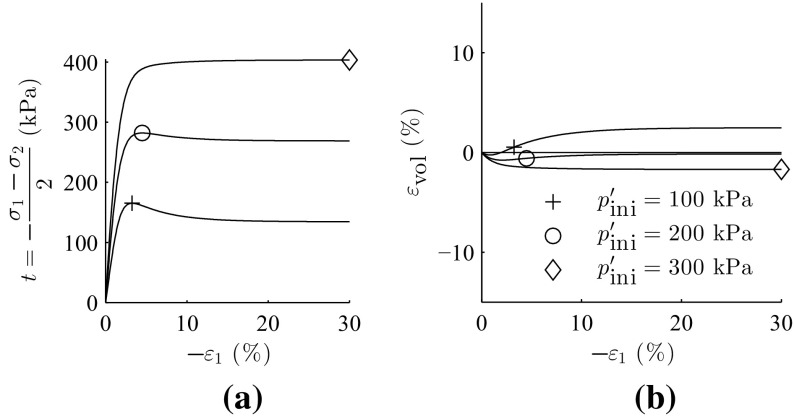
Element test with barodesy for clay, **a** stress–strain curve, **b** volumetric behavior

**Fig. 4:**
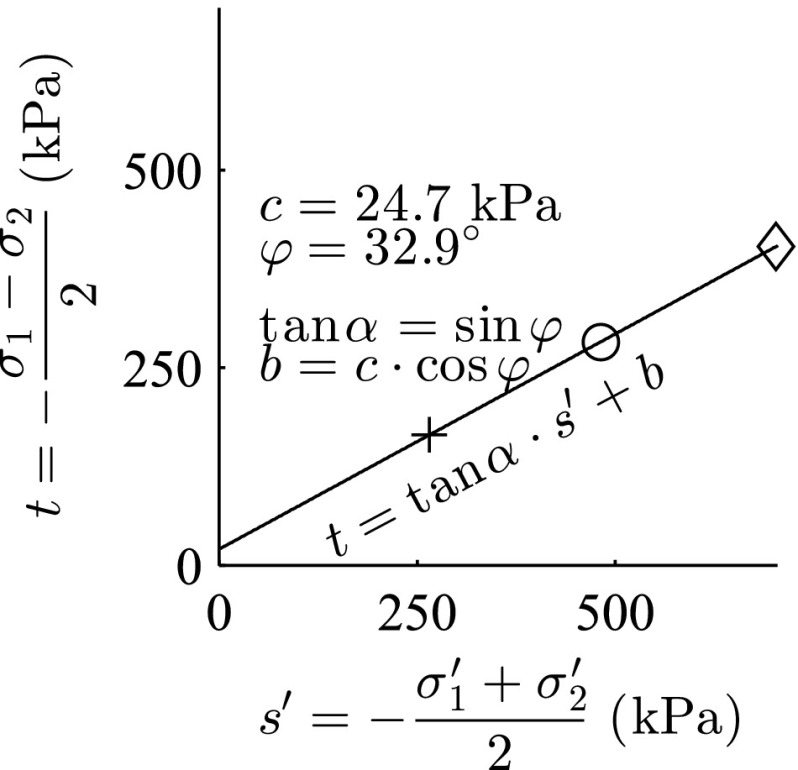
Mohr–Coulomb criterion for determination of friction angle $\varphi$ and cohesion *c*

The analytical solutions addressing the orientation of shear bands for granular materials, are based on two conventional soil parameters, friction angle $\varphi$ and dilatancy angle $\psi$ (see Sect. 6 for detailed discussion). Element simulation of triaxial test with barodesy for clay for three consolidation pressures ($p_{ini}'=$ 100, 200 and 300 kPa) were conducted and the results are presented in Fig. 3. Where $\sigma _1$ and $\sigma _2$ are the principal stress components. The acquired values achieved from the element tests were applied for determination of friction angle $\varphi$ and cohesion *c* according to Mohr–Coulomb criterion (see Fig. 4), which yields a friction angle of $\varphi =32.9^{\circ }$ and cohesion of $c=24.7$ kPa. In order to reproduce almost the same volumetric plastic strains with Mohr–Coulomb, a dilatancy angle of $\psi =7.3^{\circ }$ was assumed.

**Fig. 5:**
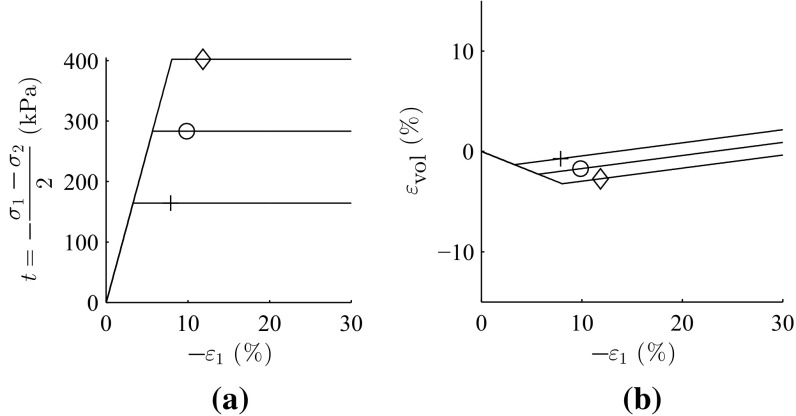
Simulation with Mohr–Coulomb for $\varphi =32.9^{\circ }$, $c=24.7$ kPa, $E=10{,}000 \text{ kPa }$ and $\nu =0.3$. **a** Stress–strain, **b** volumetric behavior for $\psi ~=~7.3^{\circ }$

Stress–strain curves and volumetric behavior with linear-elastic, perfect-plastic Mohr–Coulomb are plotted Fig. 5.

## Numerical example

### Simulation setup

**Fig. 6:**
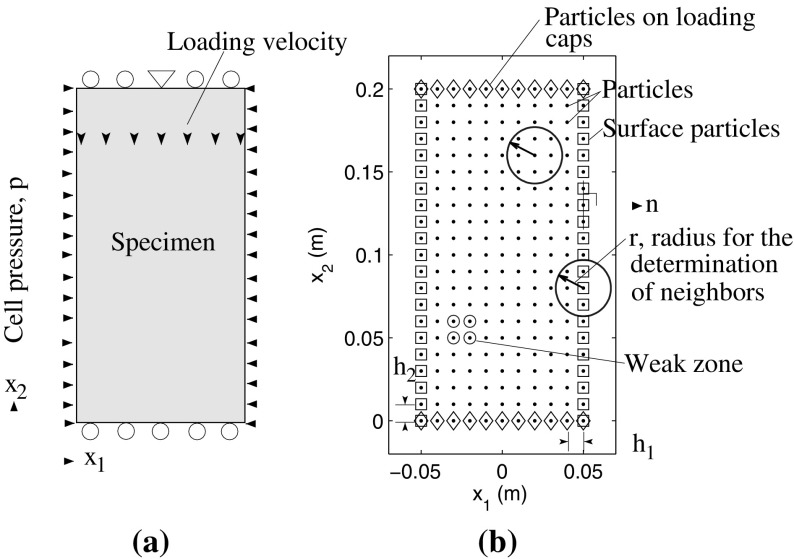
Illustration of **a** boundary conditions and **b** particles representing the study domain

A biaxial test can be regarded as a plane strain adaption of a triaxial test, to show shear localization in a 2D numerical setup. The deformation is driven by two lubricated loading caps on the top and the bottom of the specimen to allow lateral deformation. The upper cap moves downward compressing the specimen. The specimen is loaded with constant hydrostatic pressure *p* being applied to the specimen. The resulting boundary conditions are illustrated in Fig. 6a. In our simulations, the initial void ratio $e=0.45$ under a cell pressure $p=100$ kPa, corresponding to an over-consolidated clay, is adopted. Therefore, a peak in the stress–strain relationship with post peak strain softening, strain localization or formation of shear band(s) in the biaxial test simulation are to be expected.

The particle configuration for the study domain in SPARC is shown in Fig. 6b. The surface particles are subjected to cell pressure *p*. Velocity components $v_2$ of particles on loading caps are prescribed, whereas $v_1$ is unknown. Note that in order to prevent the specimen from horizontal translation, the velocity component $v_1=0$ for the particle in the middle-top of the sample is prescribed. The radius $r=\alpha \cdot h$ is used to determine neighbors for all particles in simulations where $\alpha$ has a value of 1.5–1.7 depending on the simulations^2^. The unit vector $\mathbf {n}$ normal to the pressure surface must be determined for the computation of Eq. (8). $\mathbf {n}$ is also computed using first-order polynomials in a compacted support presented in the previous section. Note that therefore, neighbors of a surface particle consist only of surface particles.

For the FE simulation, the particles shown in Fig. 6 are used for the nodes of the mesh. The boundary conditions are the same in both simulations. For the discretization, 4-node bilinear plane strain elements with four Gaussian integration points were applied.

Two simulation examples, with and without imperfection in the specimen, are presented in the following. The imperfection is implemented by increasing the void ratio of the particles representing a weak zone (Fig. 6b) by 0.02, resulting in relatively looser state and thus lower stiffness. In FE simulations, the weak zone consists of only one element formed by the same nodes. Given a weak zone, the formation of shear bands is expected to initiate from the weak zone (Fellin et al. 2009). In order to investigate the dependency of the shear bands, simulated by SPARC, on the number of particles, three sets of simulations with initially homogeneous setup for number of particles 180, 231 and 299 were conducted. Simulation with an implemented imperfection were done only for 231 particles. All simulations are summarized in Table 2.

**Table 2:** Summary of simulations

Method	Setup	No. of particles/ nodes		
SPARC	Homogeneous	180	231	299
	Imperfection	–	231	–
FE	Homogeneous	–	231	–
	Imperfection	–	231	–

### Simulations with initially homogeneous fields

**Fig. 7:**
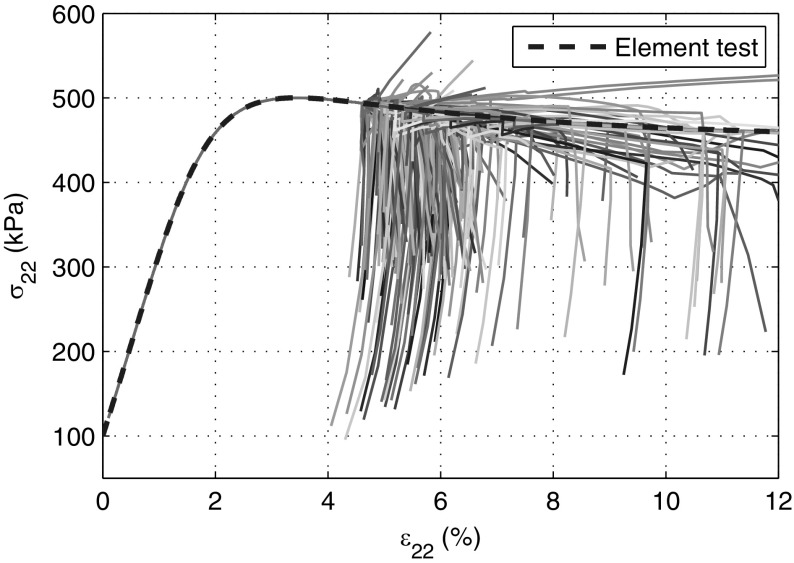
Stress–strain curves ($\sigma _{22}-\varepsilon _{22}$) of all particles in SPARC. The *blue dashed line* is the result of an element test (color figure online)

Since the initial stress field and void ratio field are homogeneous, the deformation in the sample shall be homogeneous, meaning that the stress strain curve of all particles must be identical and must overlap with the curve obtained from element test result. The constitutive model barodesy for clay is directly used to obtain the stress strain curve by prescribing the deformation matrix

24 $$\begin{aligned} \mathbf {D}=\begin{bmatrix} -1&\quad 0&\quad 0 \\ 0&\quad D_{22}&\quad 0 \\ 0&\quad 0&\quad 0 \end{bmatrix} \end{aligned}$$

and using an initial void ratio $e=0.45$ and stress state $\mathbf {T}=\varvec{1}\cdot (-100 \text{ kPa })$. $D_{22}$ is determined in each time step by satisfying the condition $\dot{T}_{22}=0$ with $\dot{T}_{22}$ obtained from the constitutive model. The fourth-order Runge–Kutta method is adopted as time integration scheme for the element test. The curve of the element test in Fig. 7 (with $\varepsilon _a=20\%$) consist of 2002 data points.

**Fig. 8:**
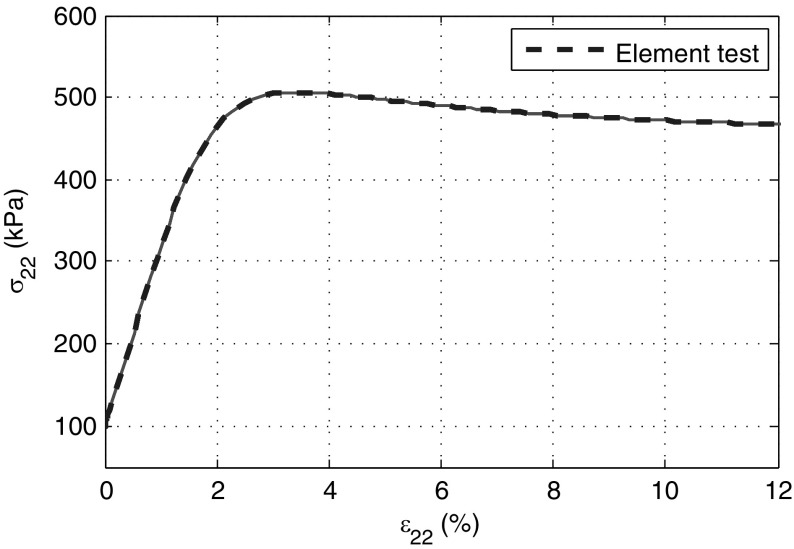
Stress–strain curves ($\sigma _{a}-\varepsilon _{a}$) of all elements in FE. The *blue dashed line* is the result of element test (color figure online)

The stress–strain curves of all particles obtained from FE and SPARC simulations are shown in Figs. 7 and 8, respectively. The SPARC simulation results show that all curves overlap with one another and with the element test curve until $\varepsilon _{22}\approx 4.6\%$ is reached. This implies that the deformation of the sample for $\varepsilon _{22}<4.6\%$ is homogeneous. Thereafter, the deformation starts to localize at particles, the localization causes numerical error and the continuation of simulation leads to the accumulation of the error. At the beginning of the simulation, $|\mathbf {D}|$ has almost the same value all over the specimen. However, the discrepancies in the $|\mathbf {D}|$ field can be recognized, even if the deformation field is relatively homogeneous. When the axial strain ($\varepsilon _{a}$) approaches $4.6\%$, strains start to localize. In the end, shear bands, revealing a ‘v’ shape, occur on the bottom of the specimen (Fig. 12c).

The void ratio field after localisation is shown in Fig. 11c, results show that contraction occurs in the whole sample at the beginning. When strain starts to significantly localize, the void ratio in the shear bands exhibit volumetric increases (see Fig. 11c). This trend is expected to occur in a dense granular sample with strain softening behavior.

In Fig. 12a, b, the deviatoric shear strain,

25 $$\begin{aligned} \gamma _s=\sqrt{\frac{2}{3} {\varvec{\varepsilon }}:{\varvec{\varepsilon }}} \end{aligned}$$

is plotted as a measure of localization of deformation.

The FE simulation, on the contrary, shows a homogeneous behavior over the whole deformation, see Figs. 11a and 12a. The evolution of the void ration *e* shows also a homogeneous compression while loading and no localization can be modeled, even at axial strain $\varepsilon _a=20\%$.

### Simulations with imperfection implemented

**Fig. 9:**
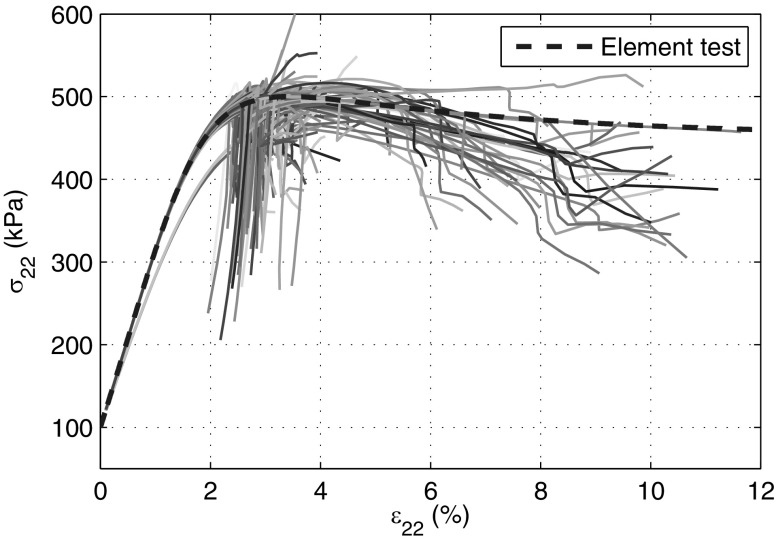
Stress–strain curves ($\sigma _{a}-\varepsilon _{a}$) of all particles in simulations with imperfection implemented in SPARC. The *blue dashed line* is the result of an element test (color figure online)

The stress strain curves in terms of $\sigma _{22}$ and $\varepsilon _{a}$ obtained by SPARC are plotted in Fig. 9. For $\varepsilon _{a}<2.0\%$ all curves except for those of particles in the weak zone are in good agreement with those of the element test curve. At $\varepsilon _{a}\approx 2.3\%$, strains start to localize significantly in a shear band initialized by the weak zone. Thereafter, strains occur mainly in the shear band. The initial shear band is followed by some other shear bands (see Figs. 11d, 12d) before the program aborts. At this point the solver cannot find any solution even with an extremely small time-steps $\varDelta t<10^{-10}$.

**Fig. 10:**
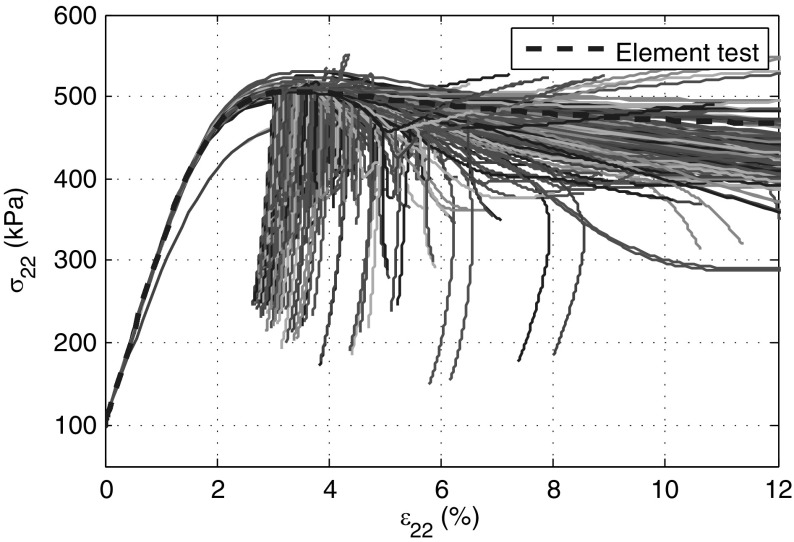
Stress–strain curves ($\sigma _{a}-\varepsilon _{a}$) of all elements in simulations with an implemented imperfection in FE. The *blue dashed line* shows the result of an element test (color figure online)

**Fig. 11:**
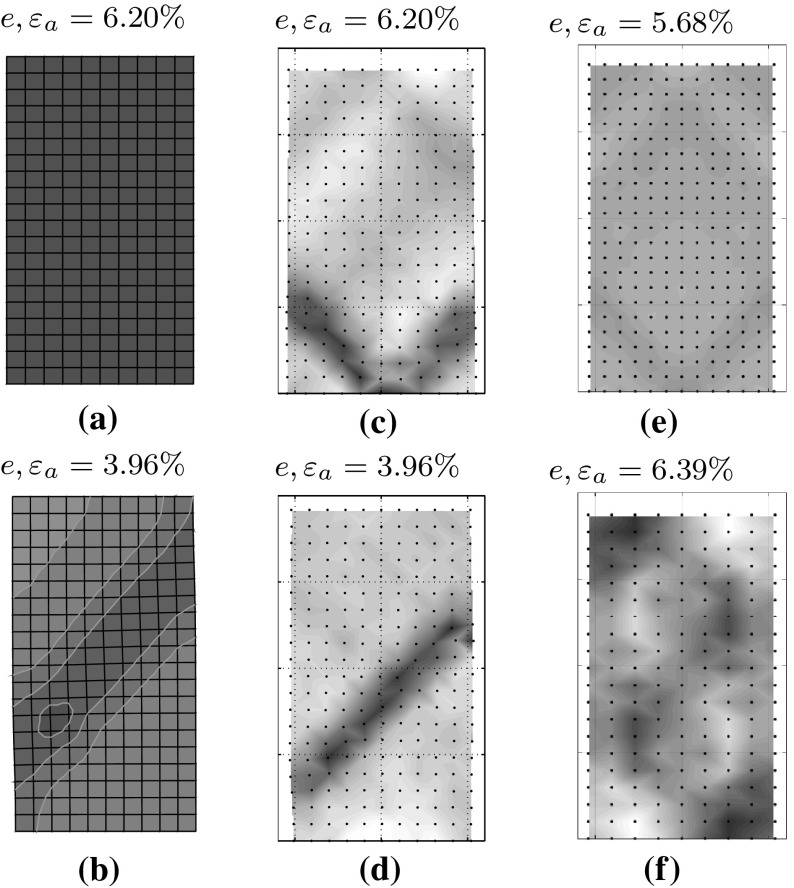
Demonstration of shear band in form of void ratio field for **a** FE with homogeneous setup, **b** FE with weak zone, **c** SPARC with homogeneous setup, 231 particles, **d** SPARC with weak zone, 231 particles, **e** SPARC homogeneous, 299 particles, **f** SPARC homogeneous, 180 particles

The void ratio field is shown in Fig. 11. Again, contraction occurs in the whole sample at the beginning. However, once the strains start to localize significantly, the changes in the void ratio can be used to demonstrate dilatancy.

The FE simulation shows comparable results. Deformations are homogeneous until the peak. Strain localization occurs at about $\varepsilon _{a}\approx 3\%$, which is larger than $\varepsilon _{a}\approx 2.3\%$ by SPARC and closer to the peak of the element test result (Fig. 10).

**Fig. 12:**
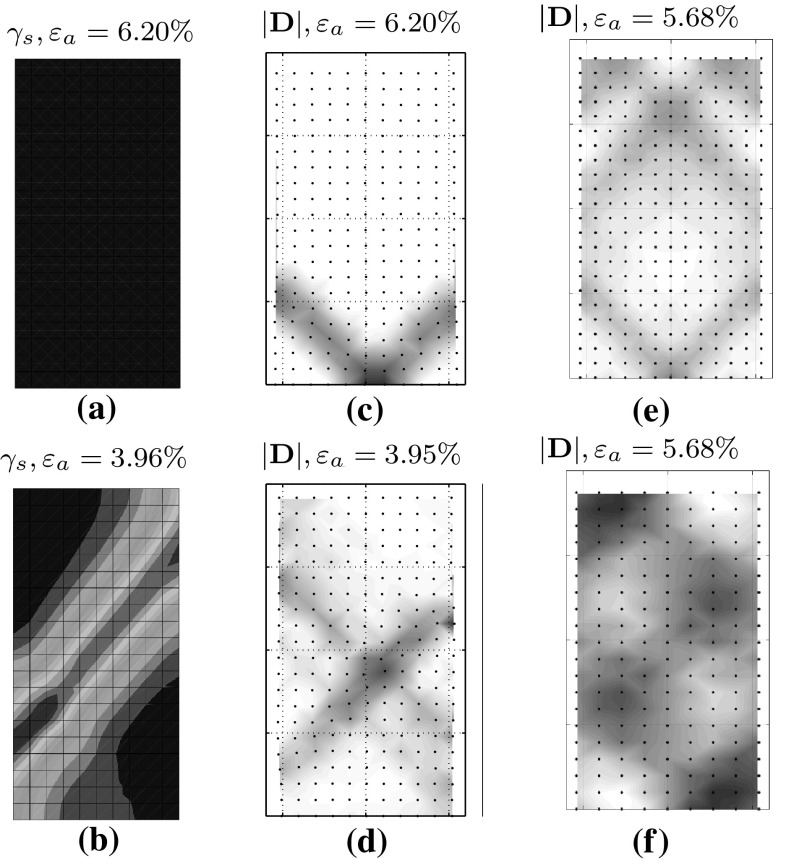
Demonstration of shear band in form of shear strain $\gamma _s$ field for **a** FE with homogeneous setup, **b** FE with weak zone and $|\mathbf {D}|$ for **c** SPARC with homogeneous setup, 231 particles **d** SPARC with weak zone, 231 particles **e** SPARC homogeneous, 299 particles, **f** SPARC homogeneous, 180 particles

The FE could reach a value of $\varepsilon _{a}=20 \%$ which is the standard value of the axial strain in triaxial tests. Shear bands start to occur in FE right before the peak of the stress–strain curve and almost in “X” pattern, however, this pattern does not last long and after reaching an $\varepsilon _a$ of greater than almost $4\%$ the shear strain localizes in a single diagonal band, see Figs. 11b and 12b. Although, FE was capable of reaching $\varepsilon _a=20\%$, results of axial strain at about $\varepsilon _a=4\%$ are included, so as to provide a more objective comparison.

## Orientation and thickness of the shear bands

Vermeer (1990) conducted theoretical and experimental investigations on the orientation $\theta$ and thickness of shear bands in biaxial tests, his investigations show that for fine sands, the orientation of shear bands coincides almost the Mohr–Coulomb solution $\theta _C=45^{\circ } + \varphi /2$ and for coarse sands, the Roscoe solution of $\theta _R=45 + \psi /2$ is observed. Where $\varphi$ and $\psi$ are the friction and the dilatancy angles, respectively. Investigations of Han and Drescher (1993) explain the dependency of the shear bands on the magnitude of the confining pressure. As mentioned in Han and Drescher (1993), the shear band inclination angle with respect to the minor principal stress decreases when the confining pressure increases, however, the shear strains increase. Experimental results of Han and Drescher (1993), have shown that at higher confining pressures (almost 400 kPa), the orientation of shear bands correspond better to the solution of Roscoe and the shear band inclination is in general much lower than the one predicted by Mohr–Coulomb. In our simulations, the acquired inclination angle with SPARC are about $39.8^{\circ }$ for the test with initially homogeneous sample and about $45.8^{\circ }$ for the test with implemented imperfection. As mentioned before, no shear band has been observed in simulation with FE for initially homogeneous sample, while for the sample with initial imperfection, the acquired inclination is about $46.8^{\circ }$.

As discussed in Sect. 4, a friction angle of $\varphi =32.9^{\circ }$ and dilatancy angle of $\psi =7.3^{\circ }$ can be attributed to Dresden clay. Considering the solution of Mohr–Coulomb with $\theta _C=45^{\circ } + \varphi /2$, we should be expecting an inclination of $\theta _C=61.4^{\circ }$ which is a clear overestimation for both the results of FE and SPARC.

However, the solution of Roscoe with $\theta _R=45 + \psi /2$ would predict a $\theta _R=48.6^{\circ }$ which seems to be a more realistic estimation both for FE and SPARC.

As for the thickness and inclination of the shear bands acquired by FE methods using a Hypoplastic constitutive model, Tejchman and Wu have shown in Tejchman and Wu (1996) that the inclination and thickness of the shear band are dependent on the spatial discretization. In a further investigation, Tejchman and Bauer (1996) benefit from the results of an extension of the Hypoplastic model for polar continuum with a characteristic length, the so-called mean grain diameter $d_{50}$. Their results show, the thickness of the shear band is the same for a fine and coarse mesh. In order to realistically simulate the thickness of the shear zone within a polar continuum, the size of the finite element should be smaller than $5\cdot d_{50}$. As it can be seen in Figs. 11b and 12b, the shear band acquired for FE calculations show an inclination of almost $52^{\circ }$ and a width of 0.028 m which is almost equal to the width of 4 elements.

In Figs. 11c, e, f and  12c, e, f, results of SPARC for a homogeneous setup and for different number of particles are presented. As can bee seen, for the lower number of 180 particles, shear bands are not clearly formed and deformation seems to localize on the two corners of up-left and down-right. This phenomenon can be so explained, that in case of homogeneous setup, the shear bands appear as a result of the accumulated error in each time-step, and with less number of particles, the accumulation stays smaller which can lead to later occurrence of shear bands or no meaningful occurrence of shear bands.

For more number of 231 particles, the shear band has a “v” shape at the middle bottom of the specimen and has a thickness, containing almost 6 particles (see Fig. 12c), while for more number of 299 particles (see Figs. 11e, 12e), the shear band is not as thick as by 231 particles and contains almost 4 particles. Furthermore, for 299 particles, shear bands have a symmetric shape not only along $x_1$ axis, but also along $x_2$ axis.

As for the inclination, the acquired shear bands for more particles (299) have a slightly larger angle as those acquired for 231 particles which fits better to the solution of Roscoe.

From the acquired symmetric shape, and the larger inclination angle, it can be deduced that for SPARC, the same as for FE methods, the denser the particles, the better the shear bands can be simulated.

## Concluding remarks

In this paper, we have shown that linear approximation method used in SPARC can model the formation of shear bands, with or without implemented imperfection. The comparison with simulation result using the Finite Element method indicates that both models can simulate shear bands and strain localization when an initially weak zone has been implemented, however, FE is not capable of building any shear band starting from homogeneous conditions. As Abaqus uses the weak formulation, the equilibrium is not fulfilled for every single integration point, but for the whole problem. Starting from a homogeneous field leads to a homogenous solution for the whole field and for all all calculation steps. As a result, no shear bands can develop. SPARC uses the strong formulation to solve the differential equations. Therefore, the equilibrium is fulfilled at every single particle with a prescribed tolerance. SPARC is capable of simulating shear bands even when the specimen has an initially homogeneous setup, this is due to the numerical inaccuracy and error accumulation in the domain. However, this corresponds the reality for experiments with homogeneous setup, that shear bands still occur.

Simulations with different number of particles with SPARC have demonstrated that the density of particles also plays a role in shape, thickness and orientation of shear bands and the denser the particles, the better the shear bands can be reproduced in the framework of SPARC.

Results of simulations with SPARC and FE with implemented imperfection and the same number of particles or nodes, have shown that the inclination and shape of shear band from both methods is comparable and almost similar. One can deduce that simulation of shear bands is not strongly dependent on the applied numerical method, but mainly on the density of particles, mesh size, or the implemented constitutive method, as discussed e.g. in Sect. 6 for constitutive models, developed for polar continuum.

The idea of using the velocity gradient and the stretching tensors in the framework of SPARC, provides a more comprehensive structure for dealing with large deformations such as strain localization in granular materials. Furthermore, it offers the advantage that the stretching tensor can be directly used as an input of many constitutive models such as hypoplasticity and barodesy. As for further numerical investigation of shear bands, it is aimed to develop SPARC for saturated conditions and conduct the same biaxial tests for hydromechanically coupled conditions and investigate the development of shear bands for both dense and loose samples and to compare the results with the experimental investigations on undrained biaxial tests.

## Footnotes

1 The superscript 0 marks a normalized tensor, i.e., $\mathbf {X}^0=~\mathbf {X}/|\mathbf {X}|\cdot \mathbf {1}$.

2
*h* = max[$h_1$, $h_2$] is the maximum spacing between particles.
